# Effects of forest conversion on soil microbial communities depend on soil layer on the eastern Tibetan Plateau of China

**DOI:** 10.1371/journal.pone.0186053

**Published:** 2017-10-05

**Authors:** Ruoyang He, Kaijun Yang, Zhijie Li, Martin Schädler, Wanqin Yang, Fuzhong Wu, Bo Tan, Li Zhang, Zhenfeng Xu

**Affiliations:** 1 Institute of Ecology and Forest, Sichuan Agricultural University, Chengdu, China; 2 Helmholtz Centre for Environmental Research-UFZ, Department of Community Ecology, Halle, Germany; 3 College of Forestry, Sichuan Agricultural University, Chengdu, China; RMIT University, AUSTRALIA

## Abstract

Forest land-use changes have long been suggested to profoundly affect soil microbial communities. However, how forest type conversion influences soil microbial properties remains unclear in Tibetan boreal forests. The aim of this study was to explore variations of soil microbial profiles in the surface organic layer and subsurface mineral soil among three contrasting forests (natural coniferous forest, NF; secondary birch forest, SF and spruce plantation, PT). Soil microbial biomass, activity and community structure of the two layers were investigated by chloroform fumigation, substrate respiration and phospholipid fatty acid analysis (PLFA), respectively. In the organic layer, both NF and SF exhibited higher soil nutrient levels (carbon, nitrogen and phosphorus), microbial biomass carbon and nitrogen, microbial respiration, PLFA contents as compared to PT. However, the measured parameters in the mineral soils often did not differ following forest type conversion. Irrespective of forest types, the microbial indexes generally were greater in the organic layer than in the mineral soil. PLFAs biomarkers were significantly correlated with soil substrate pools. Taken together, forest land-use change remarkably altered microbial community in the organic layer but often did not affect them in the mineral soil. The microbial responses to forest land-use change depend on soil layer, with organic horizons being more sensitive to forest conversion.

## Introduction

Soil microbes play an important role in forest ecosystems through decomposition of organic matter, carbon and nutrient cycling, humic compound incorporation into mineral soils, and linking plant and ecosystem functions [1]. Soil microbial communities are very sensitive to forest land-use changes [2]. It has been reported that different forests may select for distinct below-ground soil microbial communities [3]. Thus, forest type conversion could induce significant shifts in soil microbial community via biotic and abiotic factors, including species composition, above- and below-ground litter, and soil substrate quality and quantity, which are associated closely with soil microbial community [4, 5]. A number of studies have suggested that the structure and function of microbial communities in forest soils are affected strongly by tree species and composition, implying an important link between above- and below-ground processes [3, 6].

It is well known that boreal forests accumulate a large amount of organic material in the surface forest floor as a result of slow decomposition processes. An organic layer often includes various stages of decomposed organic matter, such as highly decomposed, septic; moderately decomposed, hemic, and minimally decomposed [7]. Compared to mineral horizons in the soil profile, they are rich in organic matter, with typically black or dark brown in color. In boreal forests, the organic layer is considered to be the most active interface where most of biological activities occur [8]. The organic layer and the mineral soil often have different substrate quality and availability for soil microbial growth and reproduction. Therefore, soil microbial communities could be significantly different between the two soil layers. Additionally, forest type conversion generally causes significant shifts in above- and below-ground litter type and production. Therefore, compared with mineral soils, microbial properties in the organic layer may be more vulnerable to forest type conversion because the organic layer is strongly controlled by litter production and decomposition.

The subalpine forests on the eastern Tibetan Plateau are typical alpine boreal forests at low latitude, with important consequences for regional carbon balance [9]. Over the last decades, natural coniferous forests on the eastern Tibetan Plateau were deforested and reforested with dragon spruce (*Picea asperata* Mast.) under national restoration programs. Currently, there are approximately one million hectares of spruce plantations in southwestern China [10]. Additionally, secondary birch forests have also regenerated in some clear-cutting lands [11]. In general, there is a thick organic layer in these forests. A large amount of soil organic matter is stored in the organic layer in addition to that in the mineral soil in the study area [8, 12]. Forest land-use change often induces significant changes in overstory and understory vegetation composition and soil physicochemical properties [10, 11], which in turn might alter soil microbial community, especially in the organic layer. However, soil microbial community in response to forest land-use change is still poorly understood in boreal forests. Therefore, in this study, soil microbial properties (e.g., soil microbial biomass, microbial respiration and PLFAs biomarkers) were assayed in two soil layers (organic layer vs. mineral soil) among three contrasting forest types (natural coniferous forest, secondary birch forest, and dragon spruce plantation). Specifically, we tested the following hypotheses: (1) forest type conversion would alter soil nutrient pools and microbial properties; (2) microbial responses to forest land-use change would vary between soil layers; and (3) variations in the soil microbial community would be correlated with the changes of nutrient pools induced by forest conversion.

## Material and methods

### Ethics statement

We received a permission from the Lixian Forestry Bureau to conduct this study in local forests in 2015. In this study, only limited soil samples were collected to study microbial properties and our work thus had negligible influences on the function of the broader ecosystem. In addition, this study was carried out in compliance with the laws of the People’s Republic of China. The research did not involve measurements of humans or animals, and no endangered or protected plant species were involved.

### Study site and sampling

This study was conducted at the Long-term Research Station of Alpine Forest Ecosystems, which is located on the eastern Tibetan Plateau, China (102°53'-102°57'E, 31°14'-31°19'). Mean annual temperature decreased from 4°C to 2°C and mean annual precipitation increased from 820 mm to 850 mm with increasing elevation from 2700 m to 3600 m.

Since 1960s, pervasive logging for commercial use has largely reduced the forest cover on the Tibetan Plateau, especially in the eastern part (Wu and Liu, 1998). Some logging areas were reforested with dragon spruce under national restoration programs. Alternatively, secondary birch forests had also regenerated in some clear-cutting lands [11]. Currently, natural coniferous forest (NF), secondary birch forest (SF) and dragon spruce plantation (PT) are the three dominant forest types due to local forest management practice. According to the local Forestry Bureau, the dragon spruce plantation and birch secondary forest chosen in this study developed from logging operations during the 1950s and 1960s. No additional practices (e.g., fertilizer and irrigation) were used in either forests type. However, to mitigate the impacts of environmental degradation, China has been implementing large-scale conservation programs, including the Natural Forest Conservation Program and the Grain for Green Program over last decades [13]. All kinds of forests in this area, including spruce plantations established initially for commercial timber, were again used for ecological services, such as conservation of biodiversity and water, flood and erosion mitigation in addition to carbon sequestration [14]. Moreover, with the rapid growth of economies and populations, human disturbances (e.g., grazing and wild mushroom collection) have been increasing in the plantation. The understory was mainly dominated by grasses in the plantation. Conversely, the understory is dominated by mosses, woody shrubs (especially dwarf bamboos) and grasses in the natural coniferous and secondary forests. The basic conditions are shown in Table 1. The soils at the three forest types are typical brown forest soils and classified as a Cambic Umbrisols according to the IUSS Working Group [15].

**Table 1:** Basic description of three forest stands.

Forest type	Age (yr)	Dominant species	Dominant understory	Coverage	Organic soil depth (cm)
NF	>150	*Abies faxoniana*	*Rosa sweginzowii*, *Fargesia spathacea*, *Cystopteris montana*, *Carex spp*	0.9	14.0±1.8
SF	~70	*Betula albosinensis*	*Fargesia spathacea*, *Parasenecio forrestii*, *Thalictrum spp*	0.8	10.4±2.0
PT	~60	*Picea asperata*	*Berberis diaphana*, *Deyeuxia scabrescens*	0.8	9.3±2.2

NF: natural coniferous forest; SF: secondary birch forest; PT: spruce plantation

In July 2015, three independent patches were established in each forest type having similar natural conditions (natural coniferous forest, secondary birch forest and dragon spruce plantation). Soil samples of the organic layer and the upper mineral soil (10 cm) were collected in each forest. The organic layer was identified from the mineral soil via its morphology (soil color, texture, and consistency) [8, 16]. Nine cores (5 cm diameter) were taken randomly from each patch and nine samples from same patch were mixed to get one composite sample. Each composite sample was passed through a sieve (2 mm diameter), and any visible living plant material was removed manually from the sieved soil. The sieved soil was kept in the refrigerator at 4°C prior to the analysis of microbial properties. A sub-sample of each soil was air-dried and ground prior to chemical analysis.

### Soil chemical analysis

Soil organic carbon (C) was measured by oxidation with K_2_CrO_7_ in an acid medium and titration of the excess dichromate with (NH_4_)_2_Fe(SO_4_)_2_. Soil nitrogen (N) was analyzed following the Kjeldahl digestion procedure. Soil phosphorus (P) was determined using the phosphomolybdenum-yellow colorimetric method. Soil pH was measured with a calomel electrode at 1:5 soil-to-water ratio. Soil microbial biomass C (MBC) and Soil microbial biomass N (MBN) were measured by fumigation-extraction method [17]. The released C and N were converted to MBC and MBN using *k*_ec_− 0.38 and *k*_en_− 0.45, respectively.

### Soil microbial respiration

Soil microbial respiration (MR) was estimated by determining CO_2_ production over a 4-week incubation period [10]. Briefly, fresh soil samples (100 g) of the organic layer and mineral layer were adjusted to 60% water holding capacity. The soil samples were incubated in 1 L jars at 20°C. Empty jars without soils were used as controls. CO_2_ production was measured 4 weeks after the incubation by using alkali absorption method. Soil microbial respiration (MR) was calculated per unit mass in the unit time for average rate.

### PLFA analysis

The phospholipid fatty acids (PLFAs) were extracted and quantified using a modified method previously described by White [18]. Lipids from 2 g of fresh soil were extracted by a one-phase extraction technique using phosphate buffer, methanol and chloroform in a 0.8:2:1(v/v/v) ratio [19]. Phospholipids were transformed by alkaline methanolysis into fatty acid methyl esters (FAMEs), which were quantified by a gas chromatograph (GCMS-QP2010 Series, Shimadzu, Japan). The fatty acid nomenclature used in this study was that described by Frostegård et al. [19]. Bacteria markers were identified by the following PLFAs: 15:0, i15:0, a15:0, 16:0, i16:0, 17:0, i17:0, a17:0, 16:1w7c, 16:1w5t, 16:1w9c, 18:1w7c, 18:00, cy17:0, cy19:0 and 20:5 [19, 20]. Polyunsaturated PLFAs, i.e., 18:3, 18:1w9c, 18:2w6, 9c and 20:1w9c, represented fungi biomass [21, 22]. The PLFAs i15:0, a15:0, i16:0, i17:0 and a17:0 were used as gram-positive bacteria markers [23, 24], and the PLFAs 16:1w7c, 16:1w9c, 18:1w7c, cy17:0 and cy19:0 were used as gram-negative bacteria markers [25].

### Statistical analysis

Two-way ANOVA was employed to analyze the effects of forest type, soil layer and their interaction on all measured soil variables. For a given layer, one-way ANOVA with Fisher’s LSD test was used to identify significant differences in soil properties among forest types. For a given forest type, Student’s *t*-tests were used to compare the differences between soil layers. To describe the similarity or dissimilarity pattern of soil microbial composition in the two soil layers of three forest types. Moreover, redundancy analysis (RDA) was used to visualize the correlations between PLFAs profiles and soil properties (e.g., C, N, P, and pH) by using the CANOCO software (version 4.5, Microcomputer Power, Inc., Ithaca, NY). The statistical tests were considered significant at the *p*< 0.05. The statistical tests were performed using IBM SPSS Statistics 20.0.

## Results

### Soil chemical properties

Soil C, N and P were 2.9–4.7, 2.0–6.3 and 1.2–2.4 times higher in the organic layer than in the mineral soils among three forests, respectively (Table 2). In the organic layer, soil C, N and P in both NF and SF were significantly higher than those in the PF. However, soil C, N and P concentrations were greatest in the SF in the mineral soil compared to the other two forest types (Table 2). There were no significant differences in C:N and C:P ratios among forest types within a given layer. However, C:P ratio was higher in the organic layer in each forest type as compared to mineral soil (Table 2). Soil pH in the mineral layer was higher for NF compared to SF and PT (Table 2). The statistical analysis revealed a significant forest type × soil layer interaction effect for N, P and pH (Table 2). Soil N and P were lower in the PT compared to NF and SF in the organic layer, but they peaked in SF in the mineral layer. The pH-values peaked in SF for the organic layer, but in PT for the mineral layer.

**Table 2:** Soil chemical properties in the organic and mineral soils of three subalpine forests.

Forest type	Soil layer	C(g/kg)	N(g/kg)	P(g/kg)	C:N	C:P	pH
NF	OL	206.46±12.27	13.98±1.06	0.59±0.05	15±1	352±33	5.10±0.22
	ML	43.61±14.27	2.23±0.49	0.25±0.04	19±2.	179±75	5.78±0.26
SF	OL	215.78±85.11	13.78±3.62	0.50±0.05	15±2	445±225	7.21±0.16
	ML	65.92±19.13	4.62±1.93	0.41±0.04	14±2	158±31	6.48±0.28
PT	OL	88.51±18.23	5.43±0.86	0.41±0.02	16±1	217±40	6.40±0.05
	ML	30.76±14.28	1.71±1.04	0.35±0.01	19±2	88±37	7.33±0.07
forest type		**	***	*	ns	ns	***
soil layer		***	***	***	*	**	ns
forest type × soil layer		ns	**	***	ns	ns	**

NF: natural coniferous forest; SF: secondary birch forest; PT: dragon spruce plantation; OL: organic layer; ML: mineral layer; The values are means ± SD, n = 3. ns: non-significant;

*: *p*<0.05;

**: *p*<0.01;

***: *p*<0.001.

### Soil MBC, MBN and MR

**Fig 1:**
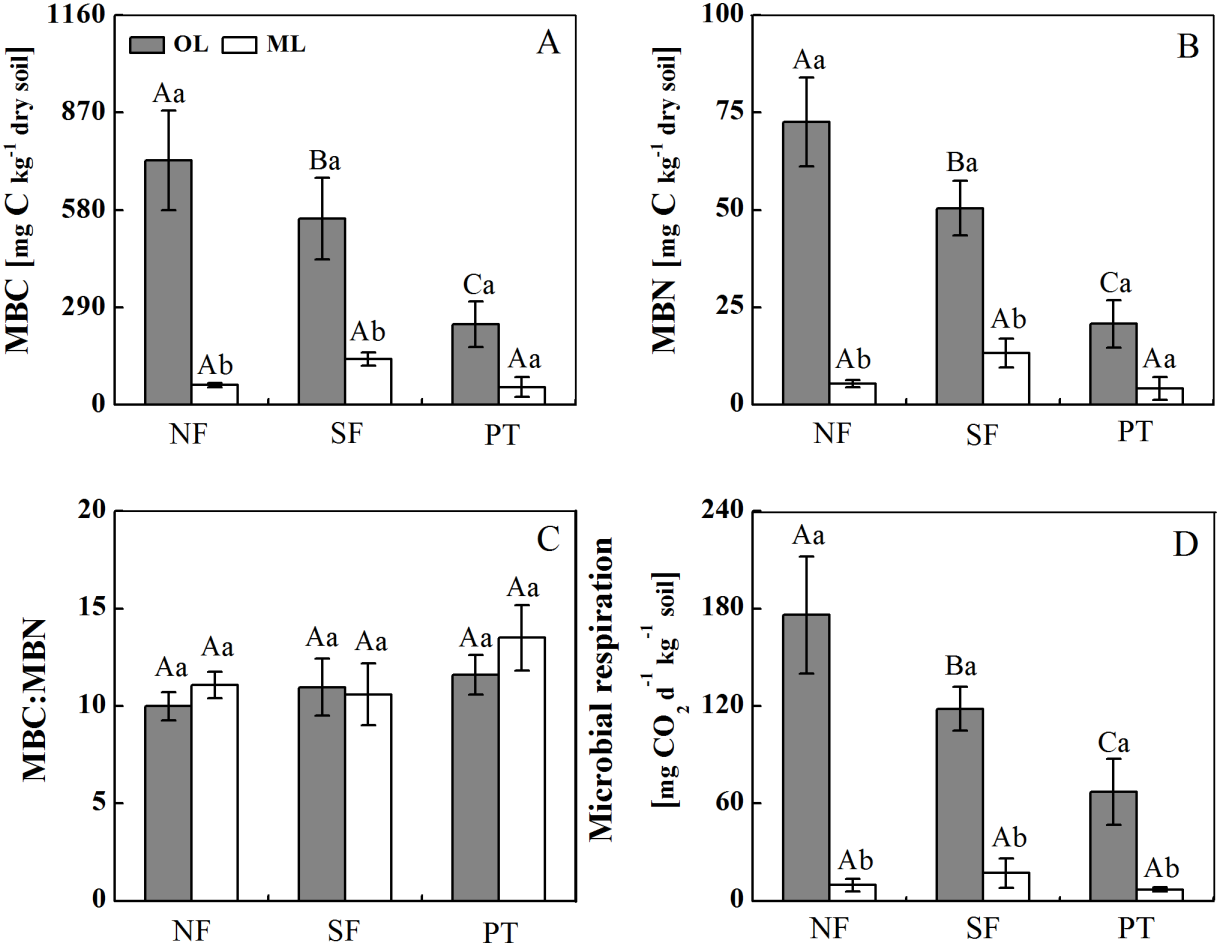
Soil microbial biomass carbon (MBC), nitrogen (MBN), MBC:MBN and microbial respiration in three contrasting subalpine forests on the eastern Tibetan Plateau. Different uppercases denote significant differences between forest types in same soil layer. Different lowercases denote significant differences between soil layers in same forest type. NF: natural coniferous forest; PT: spruce plantation; SF: secondary birch forest. The values are means ± SD, n = 3.

Regardless of forest types, soil microbial biomass carbon (MBC), microbial biomass nitrogen (MBN) and MR were significantly lower in the mineral soil than in the organic layer (Fig 1A, 1B and 1D; Table 3). In the organic layer, MBC, MBN and MR all showed a trend of NF>SF>PT (Fig 1A, 1B and 1D). However, there were no significant differences in MBC, MBN and MR among forest types in the mineral soil (Fig 1A, 1B and 1D; Table 3). Forest type, soil layer and their interaction all had significant influence on MBC, MBN and MR, but did not affect MBC:MBN ratio (Fig 1C; Table 3).

**Table 3:** Results of two-way ANOVA showing the *p* values for responses of measured variables to forest type and soil layer.

Parameters	forest type	soil layer	forest type × soil layer
MBC	**	***	***
MBN	***	***	***
MBC:MBN	ns	ns	ns
MR	**	***	**
Total PLFAs	*	***	***
Bacteria	ns	**	**
Fungi	ns	**	**
Bacteria: Total PLFAs	**	***	***
Fungi: Total PLFAs	**	***	**
Bacteria: Fungi	*	**	**
G^+^ bacteria	ns	*	***
G^-^ bacteria	ns	***	*
G^+^ bacteria: G^-^ bacteria	ns	ns	ns

MBC: microbial biomass carbon; MBN: microbial biomass nitrogen; MBC:MBN: the ratio of MBC to MBN; MR: microbial respiration; ns: non-significant;

*: *p*<0.05;

**: *p*<0.01;

***: *p*<0.001.

### Soil microbial PLFAs characteristics

**Fig 2:**
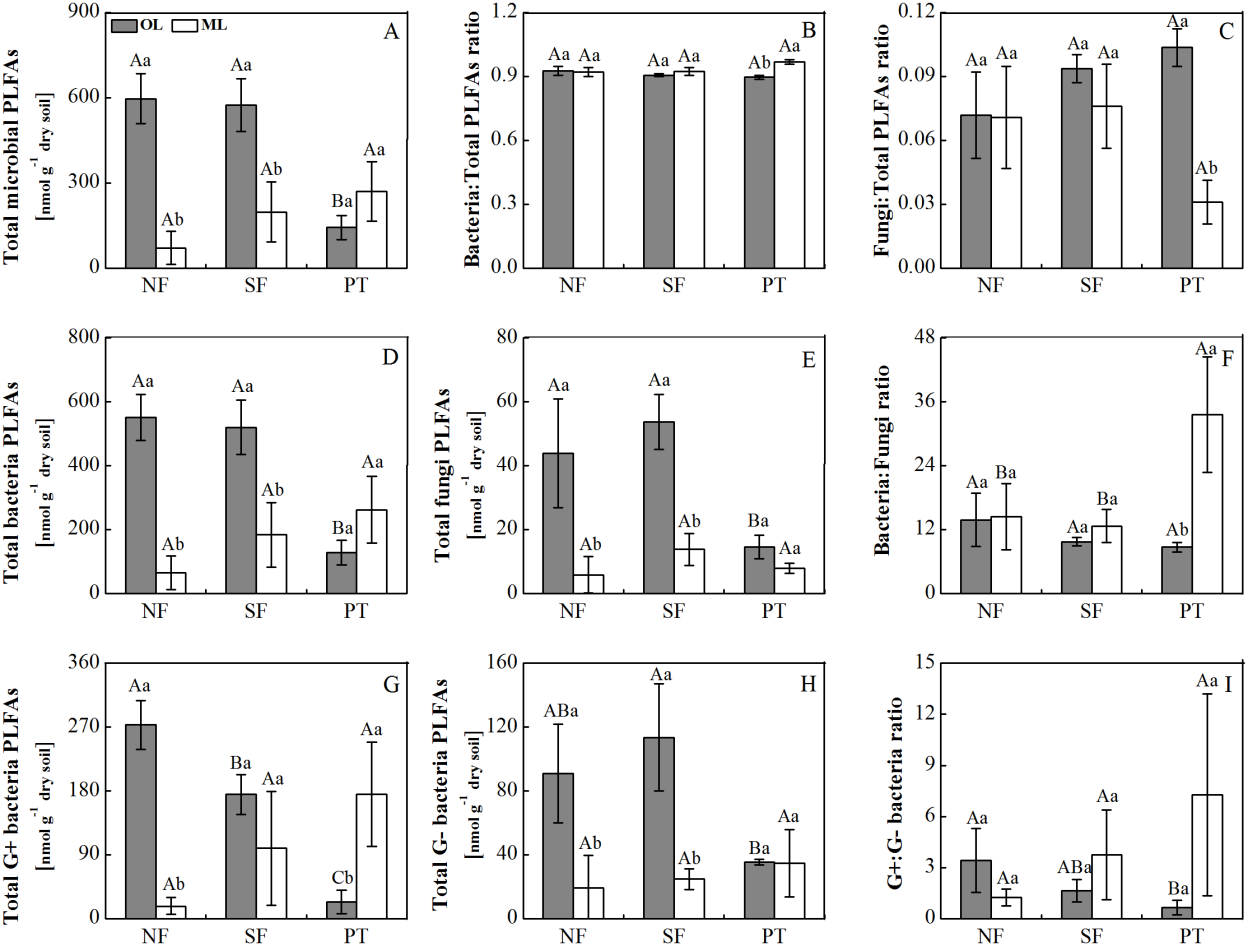
The phospholipid fatty acid biomarker contents in three contrasting subalpine forests on the eastern Tibetan Plateau. Different uppercases denote significant differences between forest types in same soil layer. Different lowercases denote significant differences between soil layers in same forest type. NF: natural coniferous forest; PT: spruce plantation; SF: secondary birch forest. The values are means ± SD, n = 3.

Forest type and soil layer generally had significant effects on PLFA variables (Fig 2; Table 3). In the organic layer, total PLFAs, bacteria, fungi, gram-positive bacteria and gram-negative bacteria were higher in both natural forest types (NF and SF) than in the PT (Fig 2; Table 3). In both NF and SF, total PLFAs, bacteria, fungi, gram-positive bacteria, gram-negative bacteria were higher in the organic layer than in the mineral soil (Fig 2A, 2D, 2E, 2G and 2H). However, in the PT forest, gram-positive bacteria and bacteria:fungi ratio were higher in the mineral soil relative to organic layer (Fig 2F and 2G). Moreover, there was significant interaction of forest type × soil layer on almost all measured microbial PLFAs parameters (Table 3).

### Correlations between soil PLFAs and physicochemical variables

**Fig 3:**
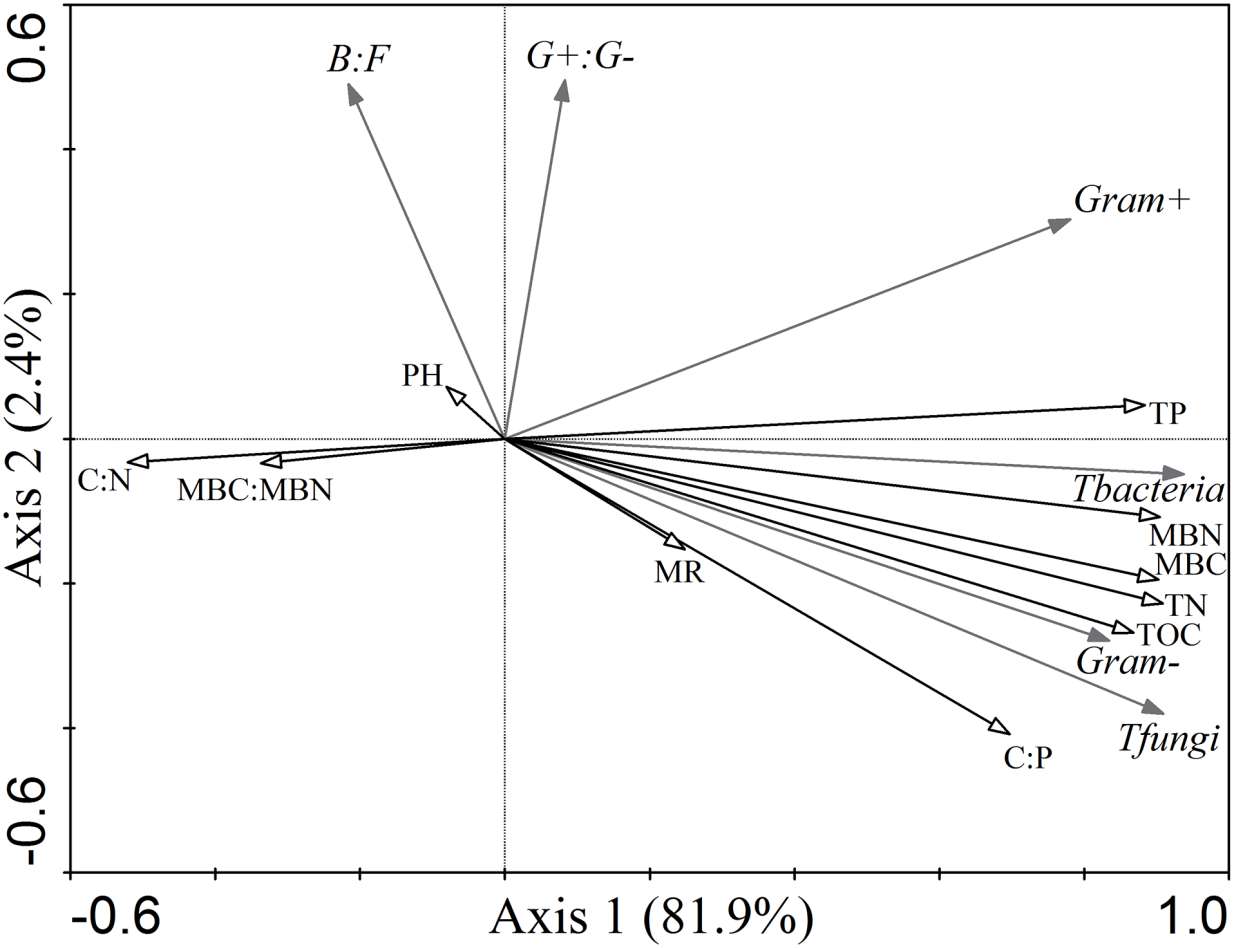
Redundancy analyses between soil microbial PLFAs and chemical parameters. Tbacteria: total bacteria PLFAs; Tfungi: total fungi PLFAs; Gram^+^: gram-positive bacteria; Gram^-^: gram-negative bacteria; B:F: the ratio of bacteria to fungi; G^+^:G^-^: the ratio of gram-positive bacteria to gram-negative bacteria. TOC: total organic carbon; TN: total nitrogen; TP: total phosphorus; MBC: microbial biomass carbon; MBN: microbial biomass nitrogen; C:N: the ratio of TOC to TN; C:P: the ratio of t to TP.

In the RDA analysis, the first and second axes accounted for 81.9% and 2.4%, respectively, of the variation in soil PLFAs (Fig 3). Soil N (67.7%, p<0.01) was the most significant explanatory variable for soil PLFAs, and thereafter the most important ones were soil MBN (67.1%, p<0.01), MBC (66.8%, p<0.01), total P (64.0%, p<0.01) and TOC (61.9%, p<0.01) (Fig 3). In addition, both soil C:P (40.2%, p<0.01) and C:N (22.2%, p<0.05) showed significant influences on soil PLFAs composition (Fig 3). It should be noted that the strong correlations between PLFAs profiles and physicochemical variables could, in part, be influenced by the auto-correlations among PLFAs biomarkers and/or explanatory variables.

## Discussion

Small changes in the microbial biomass or community structure could affect organic matter turnover and nutrient cycling [26, 27]. Therefore, soil microbial properties have been regarded as important indices reflecting the influences of forest land-use on soils [3]. It have been widely demonstrated that the composition and structure of the microbial community are strongly related to abiotic and biotic factors, such as climate factors (e.g., temperature and precipitation), soil substrate properties (e.g., C and N pools) and tree species composition and diversity [28, 29]. Thus, forest type conversion (from natural to secondary or plantation forests) may affect soil microbial biomass, diversity and community structure through altering soil substrate conditions, such as tree species, litter type and quantity, soil C and nutrient availability.

A number of studies conducted in temperate and subtropical forests have found that the conversion from natural forest to secondary or plantation forest often lowered soil microbial biomass and diversity, and altered the composition and structure of microbial communities [30, 3]. For instance, the secondary forest had higher microbial biomass as compared to larch plantation in Northeast China [31]. Moreover, total PLFAs were reduced following the conversion of natural forests to plantations in subtropical and tropical zones [32, 5]. In this study, MBC, MBN and MR exhibited a clear tendency of NF>SF>PT in the organic layer, suggesting relatively poor microbial growth and activity in the PT. There were no significant differences in total PLFAs between NF and SF; however, microbial profiles in NF and SF were higher as compared to those in PT. This observation is consistent with our previous findings where topsoil (0–15 cm) MBC and MBN were lower in PT than in NF [10]. Additionally, soil PLFAs has been demonstrated to be correlated strongly with MBC, which was in line with the result observed in this study [33].

The pattern of soil microbial profiles among the three forest types could be attributed to the differences in soil C and nutrient pools following the forest type conversion. As stated in this study, soil C, N and P pools were lower in the PT compared to NF. This was also supported by the positive correlations between microbial properties and soil C and nutrients pools. Moreover, because soil microbes primarily rely on organic C for their growth, they are profoundly controlled by any change in C input in soils [34]. In forest ecosystems, C input results mainly from the decay of soil organic matter, such as root and leaf litter, woody plant debris and root exudates. The contribution of these components is substantially dependent on overstory and understory plant species [35]. The conversion from NF to PT generally causes a considerable loss of plant species diversity, which in turn induce a significant decrease in quality and quantity of plant debris entering the soil. The Shannon-Wiener diversity index of NF (1.9–2.5) was significantly higher than those of PT (0.3–1.4) in the overstory layer [36]. Moreover, litter biomass was also greater in the NF (1.92 t ha^-2^) than in the PT (1.55 t ha^-2^) [37]. Forest land-use changes accompanied by shifts in tree species may alter quality and quantity of leaf and root litter [38], consequently affecting the substrate quality and availability for microbial growth. High quality litter with lower C:N ratio and higher N concentration can often decompose faster, resulting in rapid decomposition of organic matter relative to low quality litter [39]. Our previous studies have found that the dominant leaf and root litters (dragon spruce) in the PT have higher C:N ratios and lower N content than the equivalent litters from the SF or multi-species NF [40, 41]. Moreover, our prior studies have found that soil respiration and N mineralization rate, reflecting soil microbial activity, were also reduced markedly following the conversion from NF [9, 10]. Previous studies have also observed that soil N availabilities (e.g., dissolved organic N and inorganic N pools) were substantially greater in the NF than in the PT [42]. This may partially contribute to the microbial discrepancy among contrasting forest ecosystems because soil microbes are easier to be limited by N in rich C soil. Lastly, prior studies found that bulk density was greater in the PT relative to NF, which may also partially cause microbial degradation in the PT [10, 42].

The composition of the soil PLFAs was different among forest land-use types. The soil microbial community in the organic layer occupied different portions of ordination space in natural forests (NF and SF) and PT, indicating that the composition of the soil microbial community in NF and SF differed profoundly from that of PT. Our results found that soil microbial community was dominated by bacteria in each forest type regardless of soil layers. The bacteria groups accounted for ca. 90% of total PLFAs. Similar patterns were also observed in subtropical forests [32]. The high proportion of bacteria may attribute to high-quality substrate in subalpine forests. The lack of changes in bacteria:fungi ratios in the organic layer among three forest soils suggested that forest conversion had similar effects on the magnitude of both bacteria and fungi, resulting in similar bacteria-fungi ratios. The stability of bacteria-fungi soil food webs among different forest soils could favor subalpine forests being stable in response to forest land-use changes. However, the ratio of G^+^ to G^-^ bacteria was higher in the NF than in the PT, suggesting that the microbial bacteria community structure significantly shifted following forest conversion. The alterations in the size and structure of the soil microbial community imply low soil resource availability or high soil nutrient stress in the PT compared to NF.

In boreal forest ecosystems, there is a large amount of organic layer accumulated in the upper forest floor due to slow decomposition associated with low temperature. There are significant differences in soil substrate quality and availability between organic layer and mineral soil as a result of different rates of C input, accumulation, and turnover [43, 16]. In both NF and SF, almost all components of the soil PLFA profiles were substantially higher in the organic layer as compared to mineral soil. This may because leaf litter input provides large amounts of fresh substrates and energy for microorganisms in the organic layer, and the abundance of microorganisms is strongly related to litter input. Similar patterns were observed in other boreal forests [44]. Another source of organic matter inputs derived from root activity (e.g., root exudation, root turnover) in the organic layer could also lead to a larger microbial community in the organic horizon, as it has been reported that most fine roots are distributed in the topsoil in the fir and birch forests of southwestern China [45]. Moreover, Tibetan forests have higher nutrient pools in the organic layer relative to mineral soil [42], which may also account for the higher microbial biomass and activity of organic layer. Collectively, forest management practice profoundly alters above- and below-ground litter inputs (primary source of soil C) which regulate substrate availability and quality for soil microbial growth and activity [46]. As expected, organic layer is much more vulnerable to forest land-use change as compared to mineral soils. Prior studies have found that tree species exerted a strong effect on PLFA profiles in the organic soil, while PLFA profiles were slightly influenced by tree species in the mineral soil [47]. Our studies have also found organic soil C and N mineralization rate significantly differed among three forests but the rates of mineral soils were slightly affected by forest conversion (unpublished data). The results mentioned above clearly indicated that forest type conversion caused significant effects on soil microbial biomass and structure in southwestern China; whereas the effect of forest land-use change was strongly dependent on soil layer.

## Conclusions and implications

In summary, this study explored variations of soil microbial biomass, activity and community composition in the surface organic layer and subsurface mineral soil among three forest types. Irrespective of forest types and soil layers, soil microbial community was mainly dominated by bacteria groups in this area. Soil microbial community was higher in the natural forests (NF and SF) as compared to PT. Forest land-use change often altered microbial community profiles in the organic layer but show little or no effect in the mineral soil. The effect of forest land-use change on microbial properties depended strongly on soil layer, with organic horizon being much more sensitive to forest conversion. Moreover, microbial responses to forest land-use change could, in part, be complicated by human disturbances over past two decades.

The findings in this study have the following important implications. On the one hand, soil C and nutrient cycling could become slow following the forest type conversion as a result of low microbial biomass and activity in the PT. Moreover, it is important to note that a large amount of soil C and N were lost by deforestation in the organic layer in Tibetan forests. Therefore, protecting current natural forests is very vital to mitigate climate change. On the other hand, the differences between the organic and mineral layers in response to forest-type conversion highlight the importance and sensitivity of organic layer in Tibetan forests. Additionally, SF should be a better restoration approach in terms of soil microbial and nutrient pools as compared to PT. Future detailed work to focus on the microbial functions in the two layers would help us to better elucidate and understand the importance of soil microbial community to soil carbon and nutrient cycling in Tibetan boreal forests.
